# Conformational Reconstruction in Head and Neck Bone Cancer: Could Fibula Free Flap Become the Gold Standard Flap?

**DOI:** 10.3390/jcm14228159

**Published:** 2025-11-18

**Authors:** Gian Marco Prucher, Leonardo Gaggio, Fabrizio Neri, Fabio Astarita, Lorenzo Sani, Cristina Desiderio, Davide Allegri, Nicholas Pauro, Andrea Sandi, Anna Maria Baietti

**Affiliations:** 1 Oral and Maxillo-Facial Surgery Unit, AUSL Bologna, Via Altura 3, 40139 Bologna, Italy; 2 Oral and Maxillo-Facial Surgery Unit, Università di Parma, Via Antonio Gramsci 14, 43126 Parma, Italy; 3 Dentistry Unit, AUSL Bologna, Via Altura 3, 40139 Bologna, Italy; 4 Path and Processes Monitoring Unit, AUSL Bologna, Via Altura 3, 40139 Bologna, Italy; 5 Department of Bioengineering, University of Padua, Via Gradenigo 6/b, 35131 Padova, Italy; 6 3Dfast S.R.L., Viale della Navigazione Interna 55, 35129 Padova, Italy

**Keywords:** reconstructive surgery, CAD/CAM, mandibular reconstruction, fibula free flap, reconstruction accuracy, head and neck surgical planning

## Abstract

**Background/Objectives**: The aim of this study is to present our conformational approach to CAD/CAM planning and reconstruction of head and neck cancer defects using a single-barrel fibula free flap, where the right oral rehabilitation position and the best aesthetic recontouring are achieved through 3D conformational miniplates (3D-CMP). **Methods**: We retrospectively enrolled patients between 2024 and 2019 who underwent maxillo-mandibular cancer resection, reconstructed with single-barrel fibula free flap and 3D-CMP. All the suitable patients had a deferred procedure for implant rehabilitation. Microvascular and implant-related complications were recorded over a medium follow-up of 29 months. **Results**: Twenty-two patients were treated by demolitive and reconstructive microsurgery with 3D-CMP. Minor complications were reported in five patients. Successful flap positioning for dental rehabilitation was observed in all 16 patients. An analysis of 3D planning models and 3D post-surgery models reported a high level of accuracy. **Conclusions**: Our conformational approach, which matches a single-barrel fibula free flap with 3D-CMP, has proven to be effective in restoring aesthetics and function in our patient cohort. This approach could lead the FFF to become the gold standard for head and neck bone reconstruction compared to other bone flaps, overcoming all the limitations noted to date.

## 1. Introduction

Complex maxillofacial defects are very challenging to reconstruct. Among the available surgical options, free flaps such as the iliac crest, radial forearm, scapula, and fibula are widely used for composite defects, each with specific benefits and limitations [1,2,3,4].

The fibula-free flap (FFF), described and improved from 1975 [5,6,7,8], became the gold standard for extensive reconstructions of mandibular and maxillary defects due to its vascular reliability, bone length, and ability to support dental implants [9,10,11,12]. From the early 21st century, FFF harvesting and remodeling has improved in terms of reproducibility and precision thanks to the introduction of CAD-CAM technology [13,14,15].

The dilemma regarding the choice of a fibula positioned in the right aesthetic position or in the right prosthetic position remains. Two surgical techniques could be used to address the fibular bone width discrepancy: the double-barrel technique and the osteodistraction technique, both of which have limitations [16,17,18,19,20,21,22].

In this study, we prospectively collected data on patients who underwent head and neck cancer resection and reconstruction via fibula free flap (FFF), where the fibula bone was placed to optimize prosthetic rehabilitation, while bone contouring was restored using CAD-CAM 3D conformational mini-plates (3D-CMP).

## 2. Materials and Methods

From 2024 to 2019, we retrospectively included patients with cancer of the oral cavity involving the bone, who were selected for reconstruction via single-barrel FFF and 3D-CRP. Patients requiring reconstruction following maxillofacial trauma or benign tumors and patients not suitable for reconstruction via free flap were excluded. The selected patients followed our pre-surgical workflow: incisional biopsy, CT scan with contrast enhancement +/− PET Scan, MDT meeting, and 3D conformational reconstruction planning within 30 days of the biopsy results.

**Figure 1:**
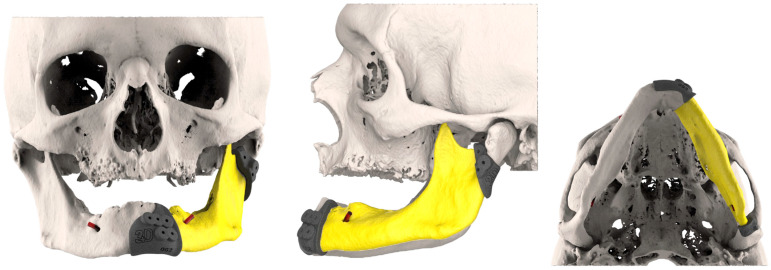
The resection plans and the volume to be removed were defined by the surgical team in collaboration with the engineers. Consequently, resection cutting guides were designed with a slight press fit solution to maintain the position over drilling.

**Figure 2:**
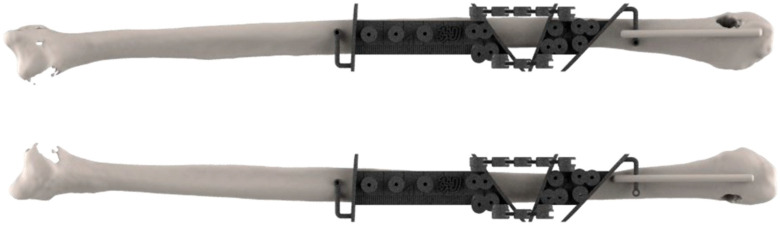
The fibula cutting guide could be swapped between the two sides: here, we show the left side on the top and the right side on the bottom.

The 3D-CRP plan requires an analysis of the DICOM CT scan data by the surgical team and the bioengineering team in a three-steps procedure:

1. Resection plan: The cutting guides are designed in accordance with the principles of radical surgical resection (Figure 1).

2. Reconstruction plan: An angiography of the leg was performed to check the peroneal vessels. A CT scan of the leg was not needed for the fibula cutting guides, which always consist of a straight 1.5 cm width polymeric guide, that is bilaterally adaptable to different kinds of bone (Figure 2). Four surgeons managed the reconstructive surgery over time.

**Figure 3:**
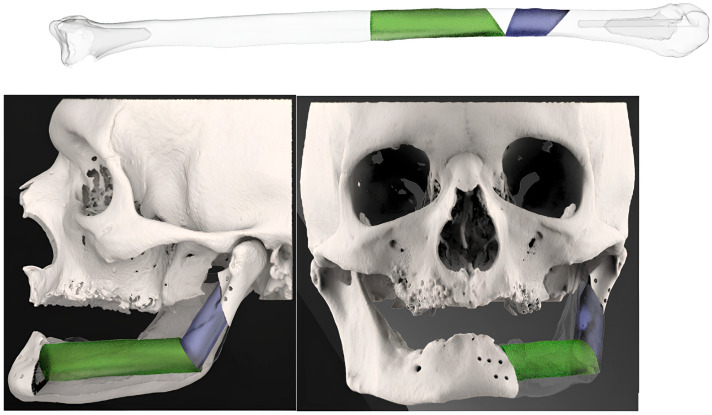
Simulation of left mandible reconstruction via fibula free flap.

Mirroring the bone defect of the original mandible-maxilla, the single-barrel fibula flap was settled with the pedicle closer to the midline in cases of mandible reconstruction and with the pedicle distally positioned in case of maxillary reconstruction. In case the mandibular angle was involved, the reconstructive plan started positioning the horizontal and vertical segment of the fibula at the same level of the gonion on the healthy side (Figure 3).

**Figure 4:**
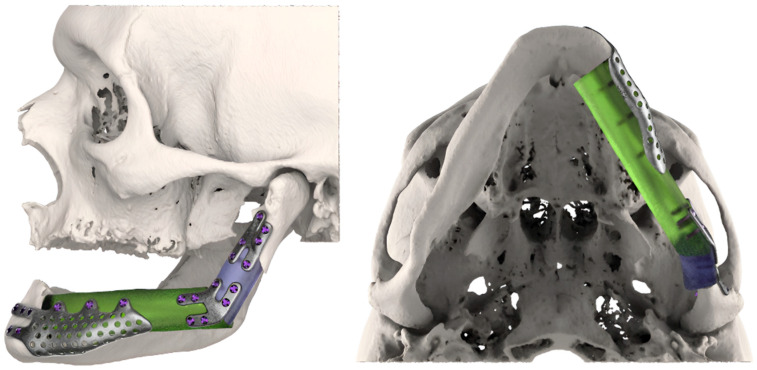
3D-conformational miniplates (3D-CMP) planning process.

After these steps, the FFF replacing mandibular body was placed at the level of the mandibular alveolar process, taking into account the best position for implants’ rehabilitation. The 3D-conformational miniplates were designed to be as short as possible to restore the aesthetic contour (Figure 4).

No condylar replacement with titanium was planned because of previous experience of glenoid fossa resorption and because, in cases of titanium plate infection, an aesthetic asymmetry would be reported after condylar prosthesis removal. If condylar reconstruction was needed, the fibula bone extremity was reshaped and stabilized into the glenoid fossa by multiple non-resorbable sutures.

**Figure 5:**
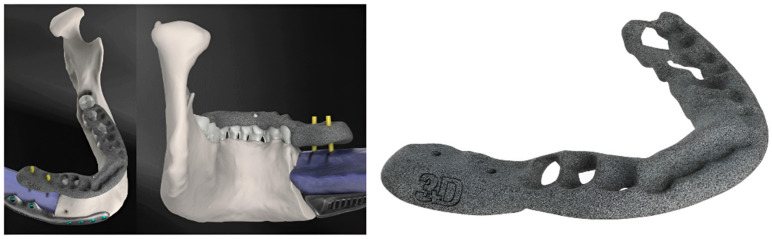
Implant positioning guide.

3. Rehabilitation plan: A custom-made 3D splint for delayed dental implant placement was planned simultaneously with the resection–reconstruction steps in order to correctly place the fixation screws between the implants (Figure 5).

The following software was used for the different steps of the 3D-CMP workflow by 3D Fast S.r.l. (Padua, Italy): Mimics 20.0, Magics 25.01, and 3-matic 12.0 (Materialise NV, Leuven, Belgium); Geomagic Freeform 2019.2.50 (3D Systems, Inc., Rock Hill, SC, USA); RealGuide 5.4.2 (3diemme—Biomet 3i, Cantù, Italy); Rhinoceros 7.0 (Robert McNeel and Associates, Seattle, WA, USA); Keyshot 7 (Luxion, Costa Mesa, CA, USA); SharkCad Pro 10 (WD Encore Software, Inc., Minneapolis, MN, USA); and SolidWorks 2020 SP2.0 (Dassault Systèmes, VÅLelizy-Villacoublay, France).

In the theater, two surgical teams, one for tumor resection and one for microsurgery, started the procedure simultaneously. The cutting guides for cancer resection and fibula segmentation were temporarily fixed in the planned positions with monocortical screws. The osteotomies were performed by a piezoelectric saw (Mectron s.p.a., Carasco (GE), Italy) or a reciprocating saw (Aesculap, B. Braun, Melsungen, Germany). The 3D-CMP was fixed to the fibula during the 3D remodeling process by mono- or bicortical screws with the free flap still connected to the vascular pedicle.

**Figure 6:**
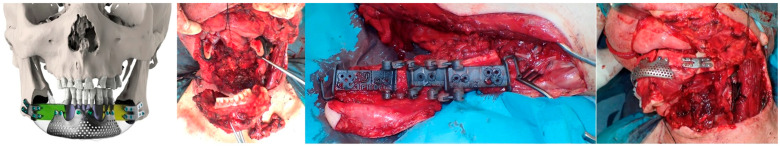
Case 1: pre-plan and intra-operative steps.

**Figure 7:**
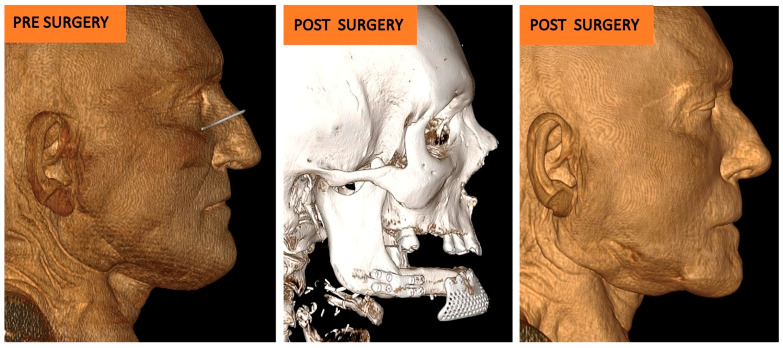
Case 1: comparative pictures of pre- and post-surgery results obtained via CT scan.

**Figure 8:**
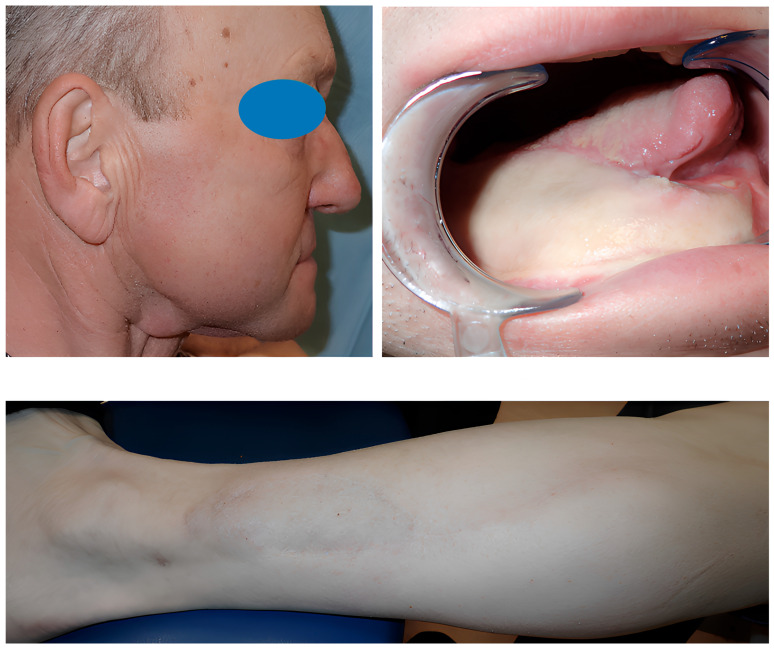
Case 1: post-surgery clinical results.

**Figure 9:**
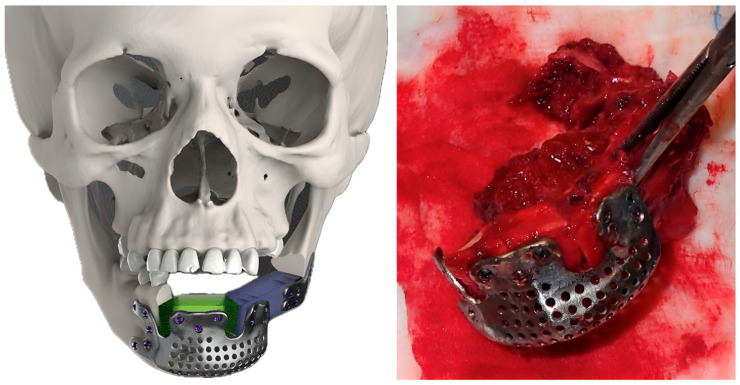
Case 2: pre-plan and FFF with 3D-CMP.

**Figure 10:**
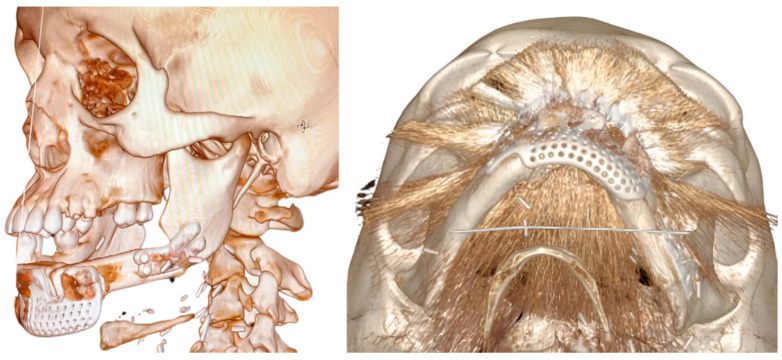
Case 2: post-surgery results obtained via CT scan.

**Figure 11:**
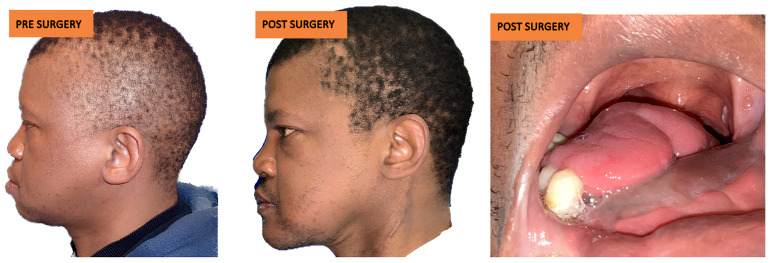
Case 2: comparative pictures of pre- and post-surgery results.

**Figure 12:**
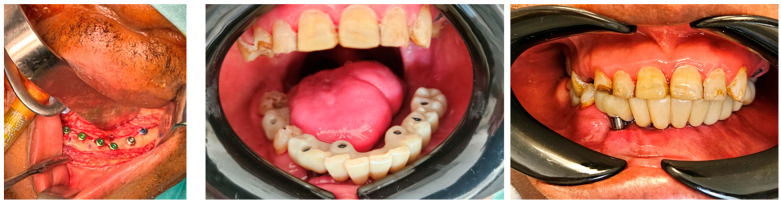
Case 2: FFF rehabilitation with oral implants and prosthesis.

After at least one year after surgery with stable results, the team performed dental rehabilitation, cosmetic surgery, and a surgical planning evaluation (Figure 6, Figure 7, Figure 8, Figure 9, Figure 10, Figure 11 and Figure 12).

The evaluation of the surgical plan accuracy was performed via a comparative analysis comparing post-surgery 3D models (from CT scan DICOM files) and 3D models of reconstruction plans.

**Figure 13:**
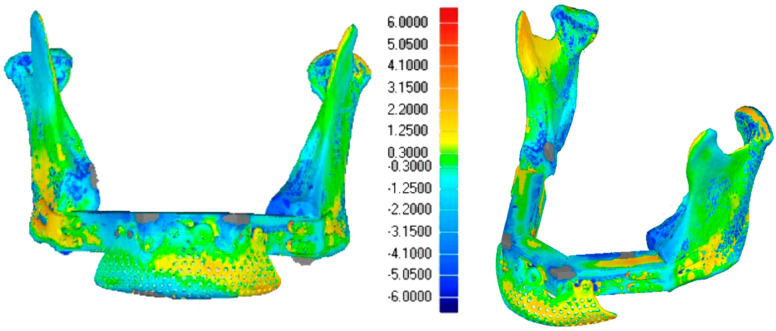
Case 1: 3D calorimetric deviation maps.

**Figure 14:**
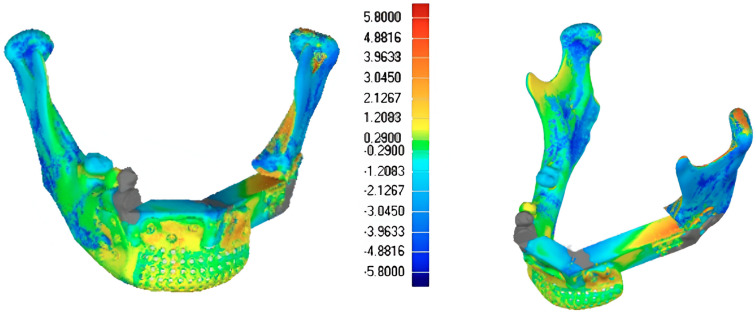
Case 2: 3D calorimetric deviation maps.

The two models were aligned using an algorithm based on a single-point best-fit registration, taking the healthy side of the mandible as a reference. Calorimetric maps were generated to highlight a millimeter-based deviation map showing postoperative discrepancies. A second evaluation was performed via comparison between the planned and the post-surgical fibula sections. The lengths of the sections were estimated to assess the degree of deviation from the surgical plan (Figure 13 and Figure 14).

These computer analyses were performed thanks to Wrap Geomegic (version 2024.3.0) and Geomegic control x (version 2024.2.0), Oqton software, USA.

Continuous variables were reported as means and standard deviations (SD) when normally distributed (assessed by Shapiro–Wilk normality test) or as medians and interquartile ranges (IQR) for non-normally distributed data. Categorical variables were presented as absolute and relative frequencies. Once the assumption of distributional normality was verified, Pearson’s correlation coefficient test was used to compare the main differences (sorted by ascending surgery date).

## 3. Results

### 3.1. Patient Characteristics (Table 1 and Table 2)

Twenty-two patients were treated (nineteen mandibles and three maxilla): seventeen were male (77.3%) and five were female (22.7%).

The mean patient age was 54 years old (range 28–77 years). Histologically, the most frequent cancer diagnosed was SCC (20 patients, 90.9%), followed by Osteosarcoma (2 patients, 9.1%). Four patients were treated over the COVID-19 pandemia, two of them in a two-stage procedure as no beds were available at that time.

The maxillary and mandibular defects treated were classified according to Brown’s classifications [23,24].

**Table 1:** Patient characteristics and treatment, sorted by ascending surgery date.

Case No.	Age/ Gender	Diagnosis/ Staging	Classification (Brown)	Adjuvant-Treatment	FFF Segments	Complications/ Implants Placed
1	67/M	Squamous cell carcinoma/St. IVa	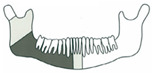 Class II		1	2 implants
2	59/F	Squamous cell carcinoma/St. III	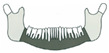 Class IV		2	7 implants
3	70/F	Squamous cell carcinoma/St. IVa	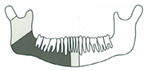 Class II	RT	2	3 implants
4	50/M	Squamous cell carcinoma/St. IVa	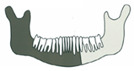 Class IV c	RT	4	Volume deficit on right cheek treated by lipofilling\6 implants
5	45/F	Squamous cell carcinoma/St. IVa	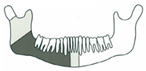 Class II	RT	2	2 implants
6	53/M	Squamous cell carcinoma/St. IVa	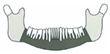 Class IV		2	6 implants
7	43/M	Squamous cell carcinoma/St. III	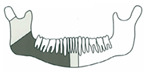 Class II		2	3 implants
8	63/F	Squamous cell carcinoma/St. IVb	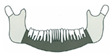 Class IV	RT	3	Dead for cancer spreading metastasis
9	48/M	Squamous cell carcinoma/St. II	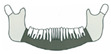 Class IV		2	5 implants
10	62/M	Squamous cell carcinoma/St. IVa	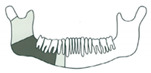 Class I		1	No implant rehabilitation
11	52/M	Squamous cell carcinoma/St. IVb	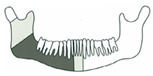 Class II	RT	2	Dead for cancer spreading metastasis
12	59/M	Squamous cell carcinoma/St. IVa	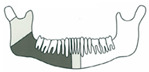 Class II		2	2 implants
13	42/M	Squamous cell carcinoma/St. IVa	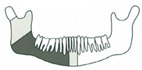 Class II	RT	2	No implant rehabilitation
14	64/M	Squamous cell carcinoma/St. IVa	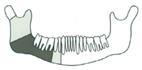 Class I		2	No implant rehabilitation/loss of FFF skin
15	38/M	Squamous cell carcinoma/St. IVa	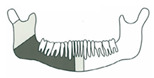 Class II	RT	2	left side cervical hematoma after 1 month\2 implants
16	47/M	Squamous cell carcinoma/St. III	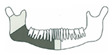 Class II		2	Osteordionecrosis and screws removal plus bone curettage/2 implants
17	66/M	Squamous cell carcinoma/St. IVa	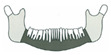 Class IV	RT	3	8 implants
18	39/M	Squamous cell carcinoma/St. II	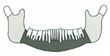 Class IV		2	6 implants
19	28/F	Osteosarcoma	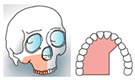 Class II d	CHT	2	Left nostril defect treated by ear cartilage graft and lipofilling\5 implants
20	57/M	Squamous cell carcinoma/St. IVa	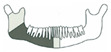 Class II		2	Volume deficit on left cheek treated by lipofilling\2 implants
21	65/M	Squamous cell carcinoma/St. IVa	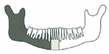 Class IIc	RT	2	No implant rehabilitation/partial loss of FFF skin
22	77/M	Osteosarcoma	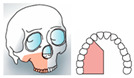 Class II b	CHT	2	3 implants

Abbreviations: RT—Radiotherapy; CHT—Chemotherapy; FFF Segments—number of segments of free fibula flap.

### 3.2. Fibula Reconstruction (Table 1)

Regarding the number of fibula free flap segments, harvested to replace of the resected mandible, 2 patients (9.1%) required one segment, 17 patients (77.3%) required two segments, 2 patients (9.1%) required three segments and 1 patient (4.5%) required four segments. The follow-up ranged from 12 months to 6 years (mean 29 months).

### 3.3. Complications

#### 3.3.1. Complications and Follow-Up

In one case, we observed segmental osteoradionecrosis and skin fistula. This patient underwent adjuvant radiotherapy 1 month after surgery and developed osteoradionecrosis on 4 of the 10 screws placed. The problem was managed by fistulectomy, removal of the screws, and bone curettage five months after the main surgery. The follow-up CT scans showed resolution of the complication.

One patient was treated a second time, one month after cancer surgery, to manage a left-side cervical hematoma. Two (9%) patients suffered from the FFF skin paddle being lost. Two patients died due to cancer metastasis at 18 and 17 months after RT.

#### 3.3.2. Aesthetic Complications

Poor aesthetic soft tissue results were reported by three patients: two of them were treated with lipofilling while the other was treated by lipofilling and cartilage graft for aesthetic improvement of the left alar rim.

### 3.4. Dental Rehabilitation

At an average time of 19 months after cancer surgery, we proceeded with rehabilitation in 16 patients by placing 64 implants by Sweden and Martina in the fibular bone, followed by dental prosthesis (Table 1). Four patients refused a second surgery for tooth rehabilitation.

The customized 3D splint designed using the 3D-CRP plan could be fitted in nine patients (56%), while the NobelBiocare tm X-Guider navigation system was used in seven patients (44%) to obtain precise placement when the teeth position changed over time. No dental implants were lost after 13 months’ mean follow-up.

### 3.5. Bioengineering Analysis

#### 3.5.1. Statistical Analysis

The results of surgical treatment were analyzed by the statistician and the bioengineering team (Table 2 and Table 3).

**Table 2:** Evaluation of fibula free flap outcomes.

Case NO.	Standard Deviation Between 3D Project Models and Post Surgery Models (mm)	Cutting Guide Segments	Project Size of FFF Segment (mm)	FFF Segment Size After Surgery (mm)	Main Difference (mm)
1	1.735	I	46	41	0
2	1.760	I	33	34	+1
		II	43	40	−3
3	2.034	I	29	32	+3
		II	44	42	−2
4	1.942	I	32	29	−3
		II	46	44	−2
		III	43	41	−2
		IV	42	41	−1
5	2.330	I	34	38	+4
		II	45	47	+2
6	2.132	I	51	49	−2
		II	37	37	0
7	1.156	I	52	52	0
		II	34	33	−1
8	2.020	I	28	26	−2
		II	44	42	−2
		III	39	35	−4
9	2.372	I	33	27	−6
		II	53	47	−6
10	1.986	I	61	60	−1
11	2.221	I	52	49	−3
		II	28	24	−4
12	1.975	I	40	37	−3
		II	35	31	−4
13	2.127	I	56	59	+3
		II	32	27	−5
14	1.845	I	51	50	−1
		II	29	28	−1
15	2.097	I	48	53	+5
		II	29	25	−4
16	2.541	I	50	44	−6
		II	53	46	−7
17	2.313	I	27	23	−4
		II	44	42	−2
		III	39	36	−3
18	2.344	I	24	21	−3
		II	51	48	−3
19	1.937	I	26	22	−4
		II	25	24	−1
20	1.910	I	27	31	+4
		II	72	72	0
21	1.429	I	54	52	−2
		II	60	60	0
22	1.826	I	26	27	+1
		II	29	28	−1

Abbreviations: SD: FFF: Fibula Free Flap segment.

**Table 3:** Distribution of FFF segments compared to patients.

FFF Number of Sgments	Number of Patients	Total FFF Sgments for Each Type
1	2 (9.1%)	2 (4.3%)
2	17 (77.3%)	34 (74%)
3	2 (9.1%)	6 (13%)
4	1 (4.5%)	4 (8.7%)
total	22	46

Pearson correlation test was performed to determine the absolute deviation between the expected and the real intervention; the standard deviation (SD) showed no significant correlation (*p*-value > 0.05) with the sequence of patients treated.

The SD analysis for patients with one fibula segment, two fibula segments, and more segments (including patients with three and four fibula segments), returned a coefficient close to zero with non-significative confidence regarding the interval values.

The analysis of the differences showed opposing trends, although none were statistically significant.

#### 3.5.2. Alignment Precision

Across all cases, the average distances between the fibula segments size after surgery and the projected fibula segments size show a main difference (mean (SD)) of −1.54 mm, with most values being negative, indicating slight under-adjustments. In general, all fibula segments demonstrated a positive correlation, although with lower intensity.

An average rate of 2.09 fibula segments was used for each patient. The standard deviation across cases suggests a variation in the degree of alignment precision, without statistical correlation.

#### 3.5.3. Morphological Restoration

These results show no differences between different surgeons and no correlation between the improvements in surgical skills over time and the management of the cutting guides.

The Standard Deviation between the 3D project models and post-surgery models (mean (SD)) was 2 mm, showing adequate restoration of the mandibular and maxillary morphology (Figure 9 and Figure 14).

## 4. Discussion

CAD/CAM technology in head and neck reconstructive surgery has reduced executive time [13,25,26], translating the simulation into reality very accurately [15,25,26]. Customized plates are able to improve structural stability [27] and minimize ischemia-related complications [13]. As reported in other studies, we confirmed the reproducibility of CAD/CAM surgery. The statistical analysis shows we obtained a high level of accuracy between fibula segments and CAD-CAM planning, even when this was performed by different surgeons without previous experience in cutting-guided surgery and in cases with higher morphological complexity. In our experience, a larger cutting-guide slice could improve the precision of the cut and better support the blade over the segmentation process, avoiding an inner rotation correlated with the under-adjustments reported in our series.

We preferred the use of a customized 3D miniplate (3D-CMP) for fibula flap fixation, because miniplates reduce the incidence of complications compared to reconstructive plates, particularly in terms of plate exposure and osteoradionecrosis [28,29]. Moreover, we never replaced the condyle via titanium prosthesis, but we remodeled the fibula bone into the glenoid fossa.

Our patients presented with different teeth positions after an average time of 19 months after surgery in 44% of cases. The absence of tooth/soft tissue support after cancer resection and the biological mechanism of surgery-mediated acceleration of tooth movement could lead to a change in position [30]. A secondary, delayed 3D planning could be considered to build a splint for implant placement a few days before implant surgery, in case no navigation-guided surgical systems are available.

Applying these techniques to our patients led to a low rate of postoperative complications and to no flap failure or loss of dental implants to date. Starting from these evaluations, the idea of restoring aesthetics and function at the same time through single-barrel FFF and 3D conformational miniplates (3D-CMP) has been positively confirmed.

Thanks to our reconstructive technique, we are able to restore a facial deformity, overcoming the traditional limitations associated with single-barrel FFF, with all the advantages that come with it.

The lack of a real control group represents the main limitation of our study.

The tridimensional analysis performed of the planning and the results were the strengths of our work.

## 5. Conclusions

Reconstructive surgery of the maxillofacial district should not be conceivable without 3D planning due to potential deficiencies in accuracy in the case of manual reconstruction, in contrast with the high reproducibility of cutting-guided surgery.

Our study highlights the benefits of integrating single-barrel fibula free flaps with CAD/CAM-designed 3D-CMP, offering a reliable solution for functional and aesthetic rehabilitation.

The 3D-CMP method simplifies the procedure, allowing for precise fibula positioning and occlusal alignment, improving aesthetic outcomes with reliable long-term results.

This approach could lead to FFF becoming the gold-standard flap for head and neck bone reconstruction compared to other bone free flaps, overcoming all the limitations noted to date.

Future research should focus on refining CAD/CAM protocols, optimizing plate designs, and exploring hybrid techniques to predict soft tissue changes. Long-term studies evaluating patient-reported outcomes and functional performance are essential to further validate the effectiveness of the 3D-CMP strategy.

## Data Availability

Data are contained within the article and further data are available from the authors upon request.

## Abbreviations

The following abbreviations are used in this manuscript:

3D-CMP	Three-dimensional conformational miniplates
CAD/CAM	Computer-Aided Design/Computer-Aided Manufacturing
FFF	Fibula Free Flap
SD	Standard deviation
